# Potent Innate Immune Response to Pathogenic *Leptospira* in Human Whole Blood

**DOI:** 10.1371/journal.pone.0018279

**Published:** 2011-03-31

**Authors:** Marga G. A. Goris, Jiri F. P. Wagenaar, Rudy A. Hartskeerl, Eric C. M. van Gorp, Simone Schuller, Avril M. Monahan, Jarlath E. Nally, Tom van der Poll, Cornelis van 't Veer

**Affiliations:** 1 Royal Tropical Institute (KIT), KIT Biomedical Research, Amsterdam, The Netherlands; 2 Department of Internal Medicine, Slotervaart Hospital, Amsterdam, The Netherlands; 3 Veterinary Sciences Centre, School of Agriculture Food Science and Veterinary Medicine, College of Life Sciences, University College Dublin, Dublin, Ireland; 4 Center for Experimental and Molecular Medicine, Academic Medical Center, University of Amsterdam, The Netherlands; 5 Center for Infection and Immunity Amsterdam (CINIMA), University of Amsterdam, Amsterdam, The Netherlands; 6 Department of Virology, Erasmus Medical Center, Rotterdam, The Netherlands; Federal University of Minas Gerais, Brazil

## Abstract

**Background:**

Leptospirosis is caused by pathogenic spirochetes of the genus *Leptospira*. The bacteria enter the human body via abraded skin or mucous membranes and may disseminate throughout. In general the clinical picture is mild but some patients develop rapidly progressive, severe disease with a high case fatality rate. Not much is known about the innate immune response to leptospires during haematogenous dissemination. Previous work showed that a human THP-1 cell line recognized heat-killed leptospires and leptospiral LPS through TLR2 instead of TLR4. The LPS of virulent leptospires displayed a lower potency to trigger TNF production by THP-1 cells compared to LPS of non-virulent leptospires.

**Methodology/Principal Findings:**

We investigated the host response and killing of virulent and non-virulent *Leptospira* of different serovars by human THP-1 cells, human PBMC's and human whole blood. Virulence of each leptospiral strain was tested in a well accepted standard guinea pig model. Virulent leptospires displayed complement resistance in human serum and whole blood while *in-vitro* attenuated non-virulent leptospires were rapidly killed in a complement dependent manner. *In vitro* stimulation of THP-1 and PBMC's with heat-killed and living leptospires showed differential serovar and cell type dependence of cytokine induction. However, at low, physiological, leptospiral dose, living virulent complement resistant strains were consistently more potent in whole blood stimulations than the corresponding non-virulent complement sensitive strains. At higher dose living virulent and non-virulent leptospires were equipotent in whole blood. Inhibition of different TLRs indicated that both TLR2 and TLR4 as well as TLR5 play a role in the whole blood cytokine response to living leptospires.

**Conclusions/Significance:**

Thus, in a minimally altered system as human whole blood, highly virulent *Leptospira* are potent inducers of the cytokine response.

## Introduction

Leptospirosis transmission occurs worldwide and comprises all infections caused by pathogenic spirochetes of the genus *Leptospira* [1]. Currently over 260 serovars are known, traditionally grouped into several serogroups [2]. Being spread by the urine of infected animals, the bacteria enter the human body via abraded skin, conjunctivae or mucous membranes, after which they may disseminate throughout the body. The majority of infections are thought to result in a mild illness with rather non-specific symptoms like fever, myalgia and headache. Some patients develop severe disease, which is often rapidly progressive and can be fatal in up to 70% of patients with severe pulmonary disorders [3].

The clinical picture of severe leptospirosis is dominated by hepato-renal impairment and hemorrhages. Patients usually die from septic shock with multi-organ failure and/or overt (pulmonary) hemorrhages. Pathological findings reveal widespread haemorrhaging in virtually all organs and tissues with diffuse inflammatory infiltrates [4]. *Leptospira* antigen has been identified in many organs, including lungs, liver and kidneys [4]–[6]. It is believed that leptospires migrate through intercellular junctions, however electron microscopy demonstrated bacteria within the cytoplasm, not contained in a membrane compartment [7].

Immunity against *Leptospira* depends on the production of circulating antibodies directed against serovar specific lipopolysaccharides (LPS). Interestingly, leptospiral LPS differs from gram-negative LPS in several biochemical, physical and biological properties [8]. Although crucial in the early stage infection, not much is known about the innate immune response to *Leptospira*. Several cytokines such as interferon (IFN)-γ, interleukin (IL)-12p40 and TNF-α, are released during infection [9]–[12]. A human monocytic cell line (THP-1), transfected with human CD14 was able to recognize heat-killed leptospires and leptospiral LPS through toll-like receptor (TLR)2 but not TLR4 [13] while TLR4 is considered to be the predominant receptor mediating a LPS induced cytokine response. Subsequent murine models, using either heat-killed and live bacteria or LPS, showed evidence that both TLR2 and TLR4 play a role [14], [15]. Leptospiral LPS was shown to be recognized by both TLR2 and TLR4 in murine cells [14] whereas leptospiral lipoproteins were recognized by TLR2 in murine kidney epithelial cells [16]. Mice with combined TLR2/TLR4 deficiency were found to be highly susceptible to lethal leptospirosis [15], [17], [18]. However, these previous studies might not be representative of the immune response to viable serovars in humans. Hence, in the present study we investigated the innate immune response to several viable leptospiral serovars in human blood. Killing assays and stimulation experiments were performed using a human monocytic cell line (THP-1), human peripheral blood mononuclear cells (PBMC) and human whole blood. Moreover we examined the involvement of TLR2, TLR4 and TLR5 in the cellular responsiveness to viable *Leptospira* serovars.

## Methods

### Ethics statement

All animal protocols were approved according to the Cruelty to Animals Act, 1876, as amended by European Communities (Amendment to cruelty to Animals Act 1879) Regulations 2002 and 2005. All animal protocols were reviewed by the University College Dublin Animal Research Ethics committee, approval P-42-05, and licensed by the Department of Health & Children, Ireland, license number B100/3682. All animal protocols were conducted according to Institution guidelines for animal husbandry, and in accordance with the principals of Replacement, Reduction and Refinement.

From the human blood donors written informed consent was obtained and the protocol was reviewed by the Medical Ethics Committee from the Academic Medical Center from the University of Amsterdam. This committee concluded that the Medical Research Involving Human Subjects Act does not apply on the presented study and that therefore official approval of this study by the Committee was not required.

### Bacteria and reagents

Several leptospiral strains from the WHO/FAO/OIE and National Collaborating Centre for Reference and Research on Leptospirosis, Amsterdam, The Netherlands, were used. Pathogenic reference strains: *Leptospira interrogans* serovar Bataviae strain Swart and *L.interrogans* serovar Lai strain Lai. These strains have been passaged for more than 20 years. For clarity reasons we refer to these strains as ‘culture-adapted’ throughout this paper. Recent isolates of *L.interrogans* serovar Bataviae and serovar Lai were obtained from leptospirosis patients. *L.interrogans* serovar Bataviae strain Kariadi-Satu was isolated in 2005 from an adult male admitted at the Dr Kariadi Hospital in Semarang, Indonesia, identified by cross-agglutinin absorption test (CAAT)[19] and sequencing [20] for serovar and species determination. Strain Kariadi-Satu was aliquoted and stored at –70°C shortly after isolation, i.e. after four *in vitro* passages, to maintain the integrity as human host-adapted variant of the reference strain. *L.interrogans* serovar Lai type Langkawi strain Langkawi was isolated from a Dutch hospitalized adult male tourist [21]. Strain Langkawi was aliquoted and stored at −70°C one year after isolation (approximately 15 *in vitro* passages). We refer to these two leptospiral isolates with low to moderate passage numbers as ‘host-adapted’ in this paper.

Saprophytic (non-pathogenic) reference strain *L.biflexa* serovar Patoc strain Patoc I and virulent *L.interrogans* serovar Copenhageni, designated RJ16441 were from the collection of the Veterinary Sciences Centre, University College Dublin, Ireland. Strain RJ16441 was obtained from a patient suffering from a severe pulmonary form of leptospirosis [22] and low *in vitro* passages of this isolate were passed through guinea pigs to maintain virulence [23].

Leptospires were grown in liquid Ellinghausen McCullough Johnson and Harris medium (EMJH), in house prepared [24]. For testing, 50 ml of a full grown culture was inoculated into 500 ml of EMJH at 30°C in a shaking incubator for 5–7 days.

Prior to the experiments, leptospires were thoroughly washed 3 times with RPMI 1640 (Invitrogen, Paisley, Scotland, UK) to remove possible LPS contamination from the culture medium, counted using a Helber Counting Chamber (Hawksley, Lancing, Sussex, UK) under darkfield microscopy and resuspended at a concentration of 2.5×10^9^ bacteria/ml from which further 10-fold dilutions were made in RPMI 1640. To heat-kill, washed leptospires were subjected to a 30 min treatment at 56°C prior to dilution in appropriate concentrations.

### Virulence assay by infection of guinea pigs

The virulence of leptospiral strains was qualitatively checked by i.p. injection of guinea pigs with each of the *Leptospira* strains according to the standard guinea pig model [23]. For each strain four guinea pigs were injected. Virulent strain RJ16441 was used as a positive control and the saprophytic strain Patoc I was used as negative control. For both positive and negative control one guinea pig was injected. Hartley male guinea pigs (Charles River Laboratories, UK) at 3 weeks of age, weighing 250 to 330 g were injected intraperitoneally with 10^7^ *in vitro* cultivated *Leptospira* in a final volume of 500 µl medium. Animals were monitored daily for signs of illness including weight loss and loss of mobility, and were euthanized when they appeared moribund. If no illness developed, they were euthanized on day 7 post infection. Liver and kidneys were cultured to detect the presence of leptospires.

### Evaluation of LigA expression

LigA expression of the different *Leptospira* strains was detected by Western blotting. Only fresh, virulent isolates are thought to express this protein [25]. Rabbit anti-LigA raised against the fragment called LigANI (amino acids 625–1224) and purified truncated LigA as positive control were both generously supplied by the Goncalo Moniz Research Centre, Fundacao Oswaldo Cruz Foundation, Salvador, Brazil. Leptospires were cultured and enumerated as described above, spun down at 1730 g for 30 min and then washed twice with PBS+5 mM MgCl_2_ wash buffer to form pellets containing 2×10^8^ leptospires/pellet. The pellets were resuspended in SDS sample buffer containing β-mercaptoethanol for standard SDS-PAGE under reducing conditions on 7.5% polyacrylamide gels. Following electrophoresis, the separated proteins were transferred to a nitrocellulose membrane and membranes were blocked with 4% skimmed milk in PBS 0.05% Tween 20. Subsequently membranes were probed with primary rabbit anti-LigA antibody followed by detection of positive bands using goat anti-rabbit immunoglobulin conjugated with horseradish peroxidase (Jackson ImmunoResearch Laboratories. Inc) and DAB staining.

### THP-1 monocytes

The THP-1 monocyte cell line was obtained from American Type Culture Collection (ATCC: TIB 202). Cells were cultured in RPMI 1640 containing 10% FCS (Invitrogen, Paisley, Scotland, UK), 2 mM glutamin (Lonza, Basel, Switzerland), 40 U/ml penicillin, 40 µg/ml streptomycin and 0.1 µg/ml amphotericin B (Invitrogen, Paisley Scotland UK) and incubated at 37°C, 5% CO_2_. Four days before the experiment, cells were washed with RPMI 1640/10%FCS/2 mM glutamin medium without antibiotics and antimycotics and sub-cultured in this medium. Prior to the experiment THP-1 cells were spun down and resuspended in fresh RPMI 1640/10%FCS/2 mM glutamin to approximately 2×10^6^ cells/ml. For experiments with normal human serum (NHS), filtrated, heat inactivated NHS (Sanquin, Amsterdam, the Netherlands) was added to the medium at a final concentration of 2% (v/v).

### Peripheral Blood Mononuclear Cells

Heparinized blood was collected aseptically from healthy donors without a known history of leptospiral infection and PBMC were obtained using Lymphoprep^TM^ (Axis-Shield, Oslo, Norway) according to the manufacturer's guidelines. Briefly, blood was diluted 1∶1 with pyrogen free 0.9% NaCl and carefully layered on top of the Lymphoprep solution and centrifuged 20 min at 800 g. The distinct band of Peripheral Blood Mononuclear Cells (PBMC) was harvested, washed with RPMI 1640 and resuspended in this medium to the same volume of the original blood.

### Whole Blood

Shortly before starting the experiment, heparinized blood was collected aseptically from healthy donors without a known history of leptospiral infection.

### Statistic methods

All experiments were performed in quadruplicate and repeated at least once. Data are presented as mean values with standard deviations. As all data was normally distributed, Students t-test was used to test for significance, whereas the α was set on 0.05. In case of experiments using whole blood or derivates, blood of two distinct donors was used.

### Innate immune response experiments

Fifty µl of whole blood or cell suspensions prepared as described above were put in the wells of a 96 well tissue culture plate (Greiner Bio-One GmbH, Frickenhausen, Germany, CELLSTAR 96-Well Polystyrene). Leptospires were diluted in RPMI 1640 until appropriate concentrations were reached. Final concentrations ranged from 2.5×10^4^ to 2.5×10^8^ bacteria/ml. Leptospires were added to the various cell suspensions to a final volume of 100 µl per well. As a blank, RPMI 1640 was used in all experiments. Concentrations of PBMC were equivalent to 50 µl whole blood per well (approximately 0.5×10^6^/ml monocytes). Plates were incubated for six hours at 37°C, 5% CO_2_. After incubation plates were centrifuged and supernatant was collected and stored at −70°C until further testing. To study TLR engagement, specific cell culture grade inhibitory monoclonal antibodies to human TLR2, TLR4 and TLR5 (InvivoGen, Toulouse, France, anti-hTLR2-IgA, anti-hTLR4-IgA and anti-hTLR5-IgA) were used. Control experiments were done using FSL-1 1 µg/ml, Pam3CSK4 100 ng/ml, LPS 1 ng/ml (*E.coli* ultrapure) and Flagellin 1 µg/ml (purified flagellin from *S.typhimurium*) from InvivoGen, known for their capacity to signal through TLR2, TLR2, TLR4 and TLR5 respectively. Antibodies were diluted in RPMI 1640 and added into the assay at a final optimal concentration of 2500 ng/ml, 1000 ng/ml and 1000 ng/ml respectively. Antibodies were preincubated in whole blood for 30 min prior to incubation with leptospires. Leptospires were added at a final concentration of 2.5×10^5^ leptospires/ml to mimic the physiological situation found in severe patients, where leptospires concentrations usually range between 10^4^–10^5^ leptospires/ml, occasionally higher [26], [27].

#### Cytokines assays

Levels of TNF-α in culture supernatants were determined by enzyme-linked immunosorbent assay (Biosource Europe SA Nivelles Belgium, human TNF-α CytoSets^TM^) according to the instructions of the manufacturer. IL-6 was determined using a cytometric beads array multiplex assay (BD Biosciences, San Jose, CA) according to the instructions of the manufacturer.

#### Killing assay

To evaluate the sensitivity of the different *Leptospira* strains to killing by host factors or cells, we incubated 2.5×10^6^ *Leptospira*/ml with whole blood, PBMC, THP-1 and 2% NHS from a healthy donor (without a known history of leptospiral infection, not heat inactivated) for six hours at 37°C, 5% CO_2_. Concentrations and final volumes were identical to the incubation experiments described above. Of every sample 50 µl was transferred into 450 µl EMJH supplemented with 0.02% 5-fluorouracil to avoid contamination. Numbers of viable bacteria were estimated by limiting dilution using serial ten-fold dilutions of samples. Growth was checked after 3 weeks incubation at 30°C by dark field microscopy.

## Results

### Virulence testing of *L. interrogans* strains by infection of guinea pigs

**Figure 1:**
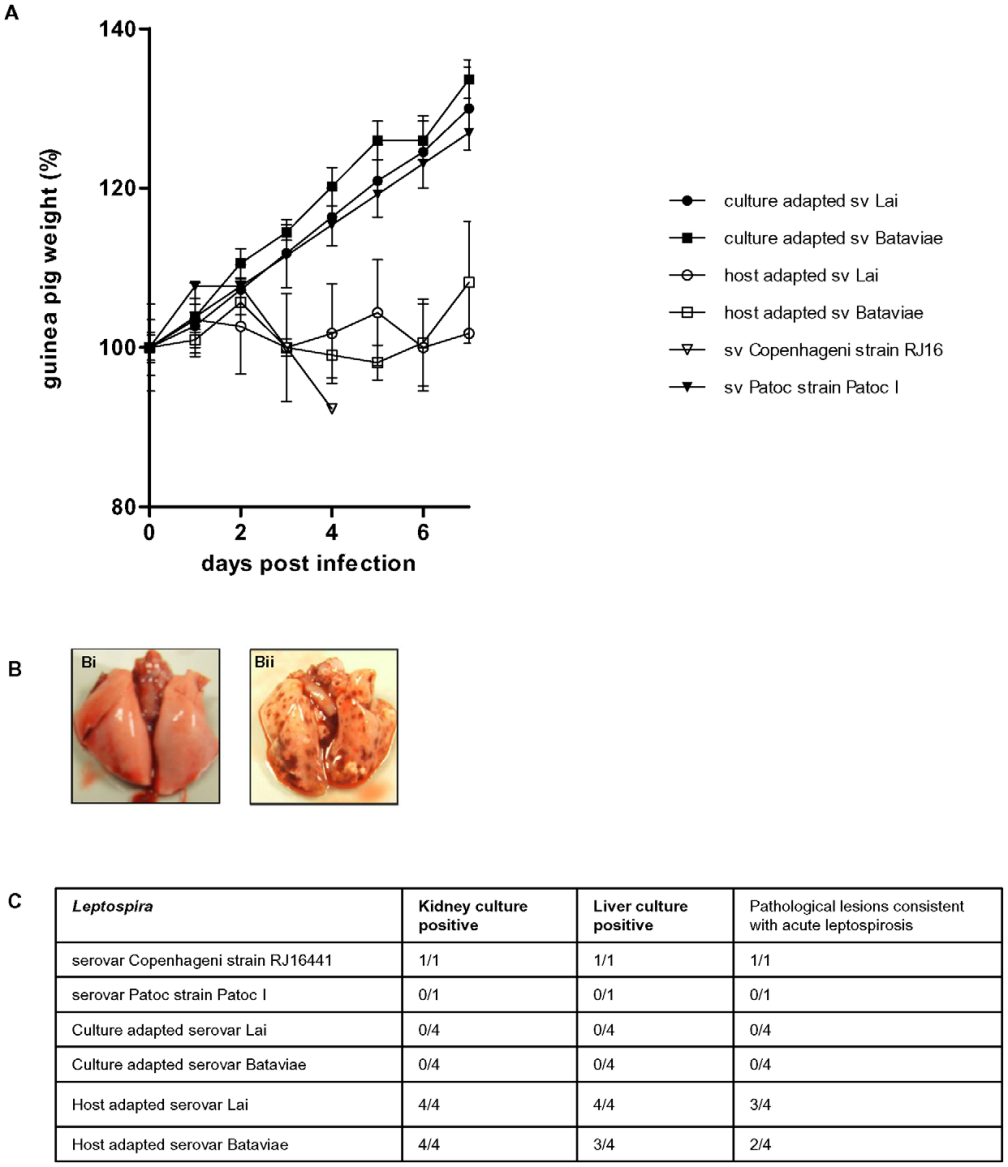
Virulence testing in guinea pigs. Hartley male guinea pigs (3 weeks of age) were injected intraperitoneally with 10^7^ *in vitro* cultivated leptospires. All guinea pigs were between 250–330 g on the day of infection (day 0). Panel A. Record of guinea pig weights. Weight is represented as percentage of average weight on day 0. Weights were recorded daily until euthanasia. Panel B. Lung gross pathology following infection with leptospires. (Bi) Typical example of culture-adapted serovar Bataviae and Lai, in guinea pig failing to induce pathology, (Bii) Typical example of host-adapted serovar Bataviae and Lai in moribund guinea pigs, showing diffuse pulmonary hemorrhages. Panel C. Culture results of kidney and liver tissue and pathology from guinea pigs *post mortem*. Pathology is considered consistent with acute leptospirosis when progressive weight loss, profound pathology, jaundice and pulmonary haemorrhage are observed.

The virulence of the *L. interrogans* strains used herein of serovars Bataviae and Lai was evaluated in the standard guinea pig model by i.p. injection of viable bacteria. The culture-adapted serovar Bataviae and Lai strains had completely lost their virulent phenotype as displayed by failure to induce weight loss or pathology and by complete clearance of viable bacteria of these strains in the guinea pig infection model (Figure 1). The host-adapted isolates are virulent based on stagnation of weight, infection and pathology (Figure 1).

### Virulence testing by detection of LigA

Western blot analysis showed that the host-adapted virulent *L.interrogans* serovar Bataviae clearly expressed LigA. The protein was only weakly detectable in the host-adapted serovar Lai, which was subjected to about ten more *in vitro* passages than Bataviae and was absent in culture-adapted and non-pathogenic strains (Figure S1).

### Different innate immune response to heat-killed and alive *L. interrogans*

**Figure 2:**
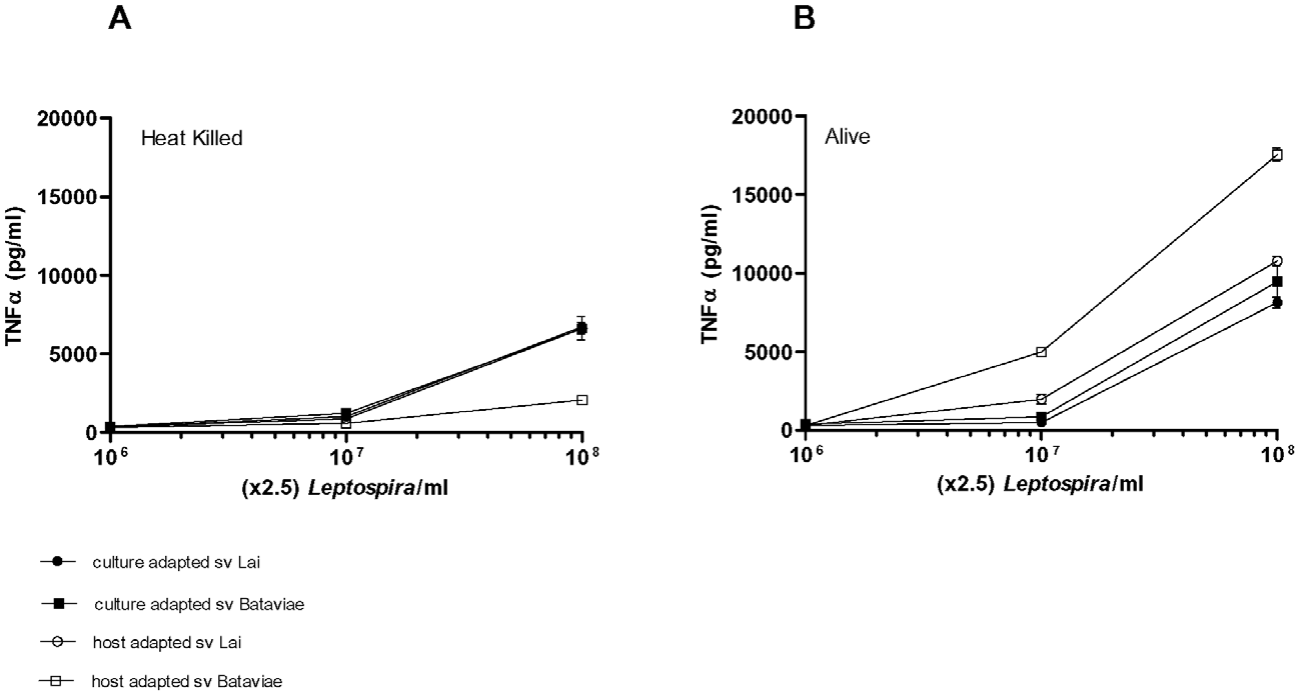
Activation of THP-1 cells with heat-killed and live leptospires. Cells were stimulated for 6 h with varying concentrations of leptospires. TNF-α concentrations were measured from cell culture supernatants. Data represent the mean values of quadruplicate experiments. Error bars represent the standard deviation (SD) of the mean. Panel A: Heat killed leptospires; panel B: Alive leptospires. The response of all blanks was below the baseline and therefore is not shown.

**Figure 3:**
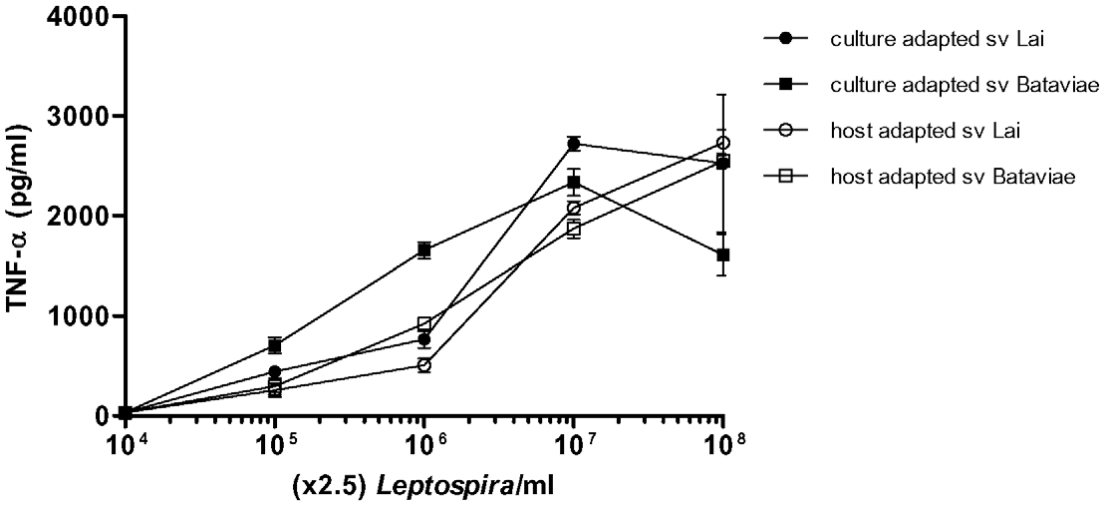
Activation of PMBC with live leptospires. Cells were stimulated for 6 h with varying concentrations of leptospires. TNF-α concentrations were measured from cell culture supernatants. Data represent the mean values and error bars represent the standard deviation (SD) of the mean. The response of all blanks was below the baseline and therefore is not shown.

Former studies investigating the innate immune response elicited by *L. interrogans* were focused on the heat-stable lipopolysaccharide moiety of these bacteria. However, since host cells encounter living bacteria during infection we compared the response of human monocytic cells (THP-1 cell line) to heat-killed and viable preparations of the culture-adapted and host-adapted strains from serovars Bataviae and Lai mentioned above. In Figure 2 the THP-1 response to the heat-killed leptospires is shown. Consistent with the previous study of Werts and co-workers [13], the heat-killed host-adapted serovar Bataviae isolate displayed a much lower potency to stimulate THP-1 cells compared to the corresponding heat-killed culture-adapted Bataviae serovar. Thus our data with serovar Bataviae are fully consistent with the notion that host-adapted virulent heat-killed *L. interrogans* are less potent innate immune activators than heat-killed culture-adapted avirulent *L.interrogans*. However, in contrast the heat-killed host-adapted serovar Lai and corresponding heat-killed culture-adapted serovar Lai displayed both the same potency as the culture-adapted serovar Bataviae. Interestingly, incubations of living preparations of the host-adapted serovar Lai and the culture-adapted serovars Lai and Bataviae resulted in a similar response of the THP-1 cells when compared to the heat-killed response with these bacteria. However, the viable preparation of the host-adapted Bataviae isolate displayed a remarkable significantly enhanced potency to stimulate THP-1 cells. It can be concluded that the virulent Bataviae strain lost a large part of its potency to activate an innate immune response upon heat-killing, and that living virulent leptospires may exert a relative potent stimulating effect on human monocytes. Since the THP-1 cell line is not exclusively an appropriate model for human monocytes we performed stimulations with human PBMC. The response to living leptospires by PBMC was more vigorous to both culture-adapted strains when compared to the host-adapted strains (Figure 3). Interestingly, in this PBMC employing test the culture-adapted Bataviae strain was most potent, while this strain displayed equal potency in THP-1 triggering as both host-adapted and culture-adapted Lai strains tested either heat-killed or alive. Similar observations were obtained with PBMC from 2 different donors. The above indicates that the relative potency to trigger innate immune responses of genetically indistinguishable virulent and avirulent leptospiral strains, as determined by *secY* sequence-based phylogeny (not shown) is largely dependent on the way of application (alive or heat-killed) and the cell type used.

### Whole blood

**Figure 4:**
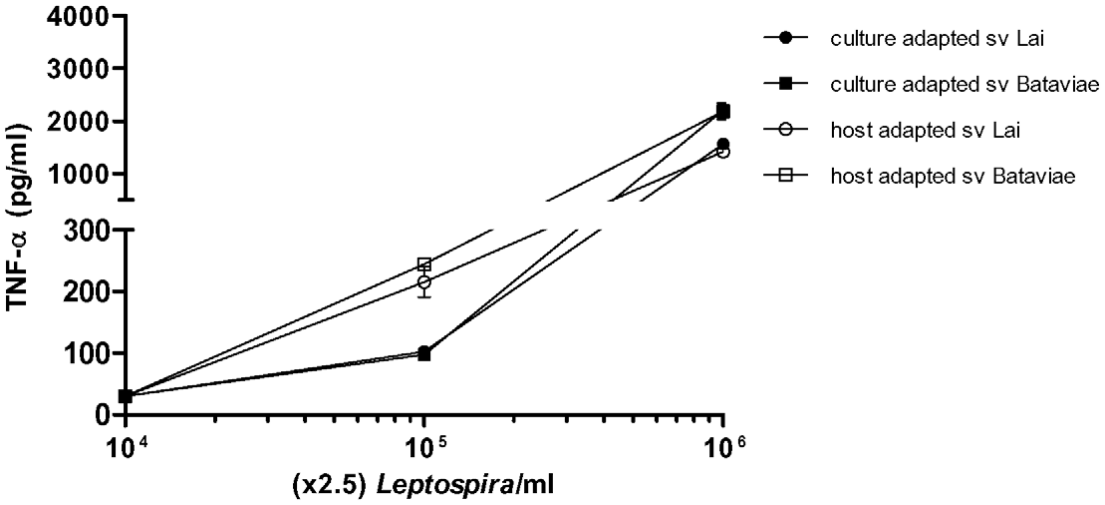
Activation of whole blood with live leptospires. Whole blood was stimulated for 6 h with varying concentrations of leptospires. TNF-α concentrations were measured from cell culture supernatants. Data represent the mean values and error bars represent the standard deviation (SD) of the mean. The response of all blanks was below the baseline and therefore is not shown.

**Figure 5:**
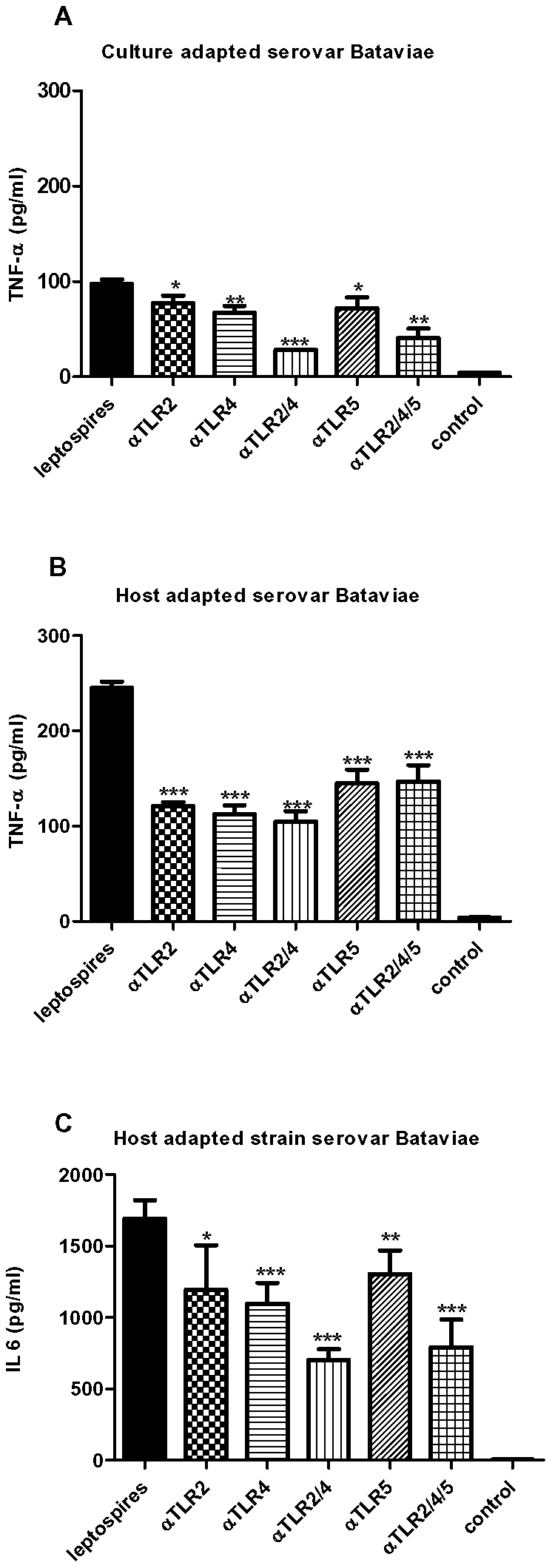
TLR2, TLR4 and TLR5 mediate activation of whole blood in response to live leptospires. Whole blood was stimulated with 2.5×10^5^/ml leptospires of host- and culture-adapted serovar Bataviae in the presence of anti-TLR2, anti-TLR4 and anti-TLR5 antibodies. Data represent mean TNF-α and Il-6 values. Error bars represent the standard deviation (SD) of the mean. Asterisks represent the significance: p value<0.001 ***, 0.001–0.01 **, 0.01-0.05 * compared to the control. Panel A: TNF-α response of culture-adapted serovar Bataviae. Panel B: TNF-α response of host-adapted serovar Bataviae. Panel C: IL-6 response of host-adapted serovar Bataviae

During the pathophysiology of leptospirosis leptospires are spread through the blood stream. To explore the proinflammatory potency of this compartment in response to virulent and avirulent leptospires we incubated the different leptospiral strains in a minimally altered whole blood assay. Similar observations were obtained in two different blood donors. Both culture-adapted strains were less potent at inducing TNF-α at low bacterial burden compared to the virulent host-adapted counterparts (Figure 4). At high, non-physiological, concentrations all strains displayed the same potency in whole blood. Similar dose-dependent responses were observed for IL-6 release (data not shown). In efforts to characterize the response in whole blood we inhibited TLR2, TLR4 and TLR5 in physiologically relevant concentrations of the virulent host-adapted serovar Bataviae [26], [27]. This involvement of different TLRs was studied using cell culture grade inhibitory antibodies from InvivoGen shown to block either human TLR2, TLR4 or TLR5 and developed for this purpose by the manufacturer. We confirmed the inhibitory capacity of the antibody preparations in control experiments (Figure S2). The majority of TNF-α production induced by virulent leptospires in whole blood could be inhibited with either anti-TLR2 or anti-TLR4 (Figure 5B). Remarkably, combined inhibition of both TLR2 and TLR4 did not further reduce the response driven by the host-adapted leptospires (Figure 5B). Blocking of TLR5 also significantly reduced TNF-α production by this host-adapted strain. Combined TLR2/TLR4/TLR5 inhibition did not result in further decrease of TNF-α release than when blocking the distinct TLRs. Blockade of TLRs showed a somewhat different pattern of inhibition on IL-6 release where anti-TLR2 was only moderately effective in decreasing IL-6 production. Combined blocking of TLR2 and TLR4 was most effective in inhibition of IL-6 release by this host-adapted serovar Bataviae (Figure 5C). When whole blood was incubated with the corresponding culture-adapted strain, the stimulation of TNF-α release by the same dose of leptospires was much lower (Figure 5A) compared to the host-adapted strain as was shown in our previous experiments. The minor amount of TNF-α induced by this non-virulent strain was slightly inhibited by blockade of either TLR2, TLR4 or TLR5, and completely prevented by combined inhibition of TLR2, TLR4 and TLR5.

### Killing of the different Leptospira strains by host factors

In whole blood the host-adapted leptospires seemed more potent in initiating an immune response than the culture-adapted leptospires, at least at moderate bacterial load, whereas isolated PBMC were more responsive to culture-adapted leptospires. Apparently, the way of exposure of leptospires to cytokine producing cells is different in whole blood compared to the exposure of leptospires in a purified mononuclear cell fraction of blood. This prompted us to test potential killing of the leptospiral strains by the different cell types, complement, or whole blood. The viability of the different leptospiral strains was evaluated semi-quantitatively by culture of serial dilutions of leptospires incubated with the different cells or blood components.

**Figure 6:**
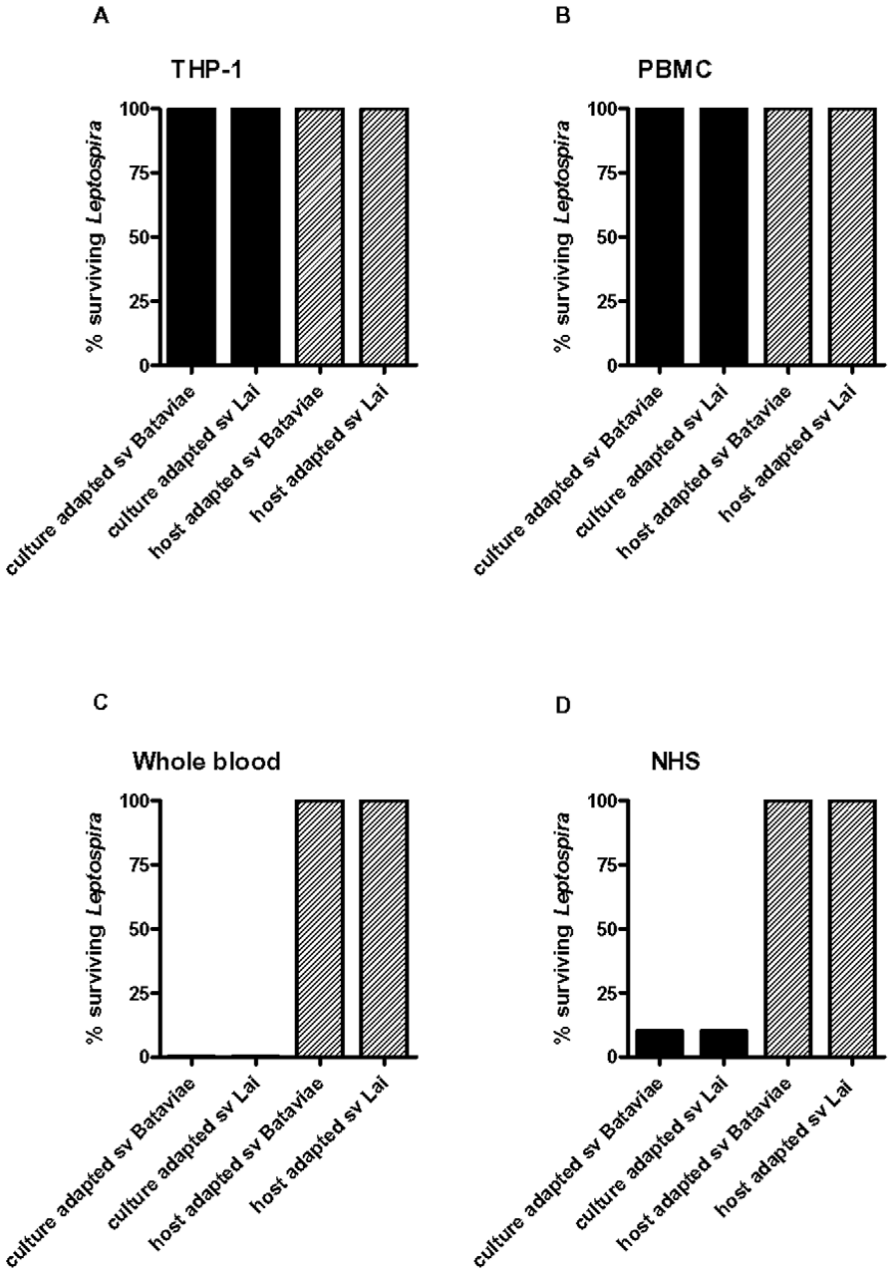
Killing of leptospires. Abbreviations: sv, serovar; NHS, normal human serum. Leptospires are incubated for 6 hours. 10-fold serial dilutions were made after 6 h in EMJH, and leptospiral growth was evaluated after 3 weeks incubation at 30°C. Culture- and host-adapted serovar Bataviae, culture- and host-adapted serovar Lai were used under the following conditions: Panel A: THP-1 cells, panel B: PBMC, panel C: whole blood, panel D: NHS

As shown in Figure 6 incubations with THP1 cells or PBMC did not affect the viability of any of the used leptospiral strains. Upon incubation with serum and whole blood the culture-adapted strains were killed, while the host-adapted strains displayed complete serum resistance and also survived the 6 hour whole blood incubation. Incubation with heat-inactivated serum did not result in killing of the culture-adapted strains (data not shown) which is in agreement with complement mediated killing of avirulent leptospires by normal serum. Consistent with the presence of host-adapted virulent leptospires in the organs of infected guinea pigs, these observations indicated that the culture-adapted leptospiral strains are efficiently and rapidly destroyed by complement activity while the host-adapted leptospiral counterparts evade complement dependent killing and also do not loose their integrity in whole blood. Obviously, the most important difference in stimulating cytokine release between host-adapted and culture-adapted strains is that the latter will be killed promptly when entering the blood and thus remain below physiological concentrations needed for detectable stimulation.

## Discussion

Leptospirosis can cause severe human disease, leading to bleeding, multi-organ failure and septic shock. The disease is caused by strains of the *Leptospira* bacteria that may differ in their LPS moiety which is used to discriminate the different leptospiral serovars [3]. Previous reports identified TLR2 as the innate receptor that recognizes *Leptospira* derived LPS as well as heat-killed whole *Leptospira* bacteria [13], [14]. Furthermore, the LPS from virulent leptospires was found to be a weaker activator of monocytes than the LPS from avirulent leptospires [13]. These findings suggested that TLR4 is not involved in the host response to leptospiral LPS, and that a potential escape mechanism of virulent bacteria occurs by concealment of the TLR2 activating LPS structure. In order to further elucidate how the innate immune system reacts to *Leptospira* we confirmed the observations with heat-killed bacteria by Werts and co-workers [13] using a monocytic cell line and comparing two culture-adapted strains representing attenuated non-virulent leptospires with their host-adapted, virulent counterparts. Indeed, when using heat-killed bacteria, the host-adapted *L. interrogans* serovar Bataviae was weakest in stimulating THP-1 cells consistent with the earlier report. However, when this host-adapted leptospiral strain was incubated alive on the monocytic cells it showed an importantly higher potency to activate immune cells in comparison to the culture-adapted strain. These experiments indicated that the host-adapted serovar Bataviae displays an important, as yet to be identified, immune activation pathway that is lost upon heat inactivation of the bacteria. However, this phenomenon did not occur when executing the experiment with the serovar Lai counterparts. This serovar displayed similar responses irrespective of its killed or viable status and for both host-adapted and culture-adapted strains. There are two possible explanations for this observation.

Firstly, differences in responses might reflect differences in expression of potential virulence factors in the two serovars. The host-adapted serovar Lai showed a markedly lower expression of LigA compared to the host-adapted serovar Bataviae, which is consistent with its higher number of *in vitro* passages [25]. Differential expression might very well have occurred for other bacterial cell components [28], [29]. In the guinea pig model, both the host-adapted serovar Lai and Bataviae escaped complement killing in the blood, enabling Lai to adapt, grow and disseminate, thus regaining its full host-adapted constitution. However, incubation time and conditions with THP-1 cells most probably did not enable such adaptation and results therefore might reflect a host interaction with a pathogen sharing more features similar to a culture-adapted than to a host-adapted form. This option is important as it suggests that virulence testing of *Leptospira* strains in animals alone may not be a reliable marker for their *in vitro* performance. Secondly, it cannot be excluded at this stage that serovar specificity does account for the differences in cytokine responses by THP-1 cells. In this respect it should be noted that, whereas no specific clinical manifestations can be associated with distinct serovars, some serovars tend to cause more serious disease than others [30]. Both explanations for different *in vitro* behaviour of the two host-adapted strains are highly relevant as these will have consequences for future interpretations of *in vitro* experiments on innate immune responses. Therefore, it is important to further investigate the role of differential gene expression and serovar specificity in innate immune responses. Differences in the outcome of *in vitro* experiments apparently is also associated with the type of host cells used in the tests because different TNF-α release profiles were observed when using PBMC compared to THP-1 cells. This observation prompted us to determine the host reaction to different leptospiral isolates in a relevant minimally altered physiological system. For this purpose we conducted whole blood incubations with viable leptospires in which we were able to show that host-adapted leptospires are as potent activators of the innate immune system as culture-adapted leptospires. Furthermore, at physiologically relevant bacterial burden host-adapted leptospiral strains of different serovars (Lai and Bataviae) displayed both an enhanced potency to stimulate the innate immune system compared to the respective culture-adapted strains. The main difference between the host-adapted isolates and their culture-adapted counterparts was the complete resistance of the host-adapted isolates to killing by whole blood and serum while the culture-adapted strains were rapidly killed. It should therefore be noted that differences found in stimulation and inhibition experiments only are of ‘academic’ interest i.e. infection with attenuated culture-adapted leptospiral strains will at the best result in the transient presence of low numbers in the blood, incapable of eliciting detectable responses. Hence further discussion focuses on results obtained with host-adapted strains. Host-adapted *L. interrogans* serovar Bataviae induced TNF-α and IL-6 release in whole blood that was partially TLR2 and TLR4 as well as TLR5 dependent. Single blockade of either TLR2, TLR4 or TLR5 prevented a large part of the TNF-α production induced by the host-adapted leptospires while combined inhibition of TLR2/4/5 did not result in a further reduction of this innate immune response. One possible explanation might be that cytokine release was stimulated in a manner that is suggestive for synergistic cooperation of TLR2, TLR4 and TLR5. We hypothesized that viable virulent leptospires may trigger the host response in whole blood by more concentrated pathogen-associated molecular patterns (PAMP's) exposed on their intact surface. Since induction of signaling by human TLR4 through LPS of *L. interrogans* is unlikely [13], [14] the observed TLR4 involvement may be caused by TLR4 activating released danger-associated molecular patterns (DAMP's) such as HmgB1 and MRP-8 [31] by host cells upon encounter of living specimen of this invasive genus of bacteria [32]. It has been suggested that TLR5 may cooperate with TLR4 in response to flagellin [33], also in whole blood [34], so another possibility is that TLR4 is engaged in a TLR5 dependent response in whole blood to living virulent *L. interrogans* (Figure 5). However, leptospiral flagella are located in the periplasm [35]. In case of virulent leptospires that are capable to survive in the blood, flagellin mediated triggering through TLR5 is not likely to occur. We argue that not all virulent leptospires may evade complement killing and that part of them are lysed resulting in exposure of flagellins in sufficient amounts to stimulate TLR5 response. It should be noted that only very low amounts of flagellin are needed to achieve a TLR5 mediated response [36]. Further research will be needed to confirm or refute this hypothesis. It must be noted that IL-6 production also indicated a role for TLR2, TLR4 and TLR5, however IL-6 production was more markedly reduced by combined inhibition of TLRs. This difference in TLR dependence between TNF-α and IL-6 production may be due to the differences in the pathways regulating gene expression and translation of these mediators (reviewed in [31]). In our experiments we observed similar cytokine responses in two donors. However, donor dependent differences in TLR2 and TLR4 pathways have been reported in a significant proportion of global populations [37]. Therefore, we can not exclude host dependent factors in cytokine responses at this stage.

To the best of our knowledge, we are the first to show that TLR2 as well as TLR4 and TLR5 play a role in the response to viable pathogenic leptospires in a human whole blood model.

Basically, human whole blood incubations with viable leptospires revealed that virulence characteristics of *L. interrogans* are associated with complete resistance to killing by normal whole blood, and not with impaired recognition by the cellular receptors of the innate immune system. These results are in line with previous work showing that pathogenic leptospires are able to survive in the non-immune host by evading, to various degree, complement-mediated killing [5], [38]. Although C3 is probably equally deposited on both non-pathogenic and pathogenic strains, the latter are able to bind factor H on their surface which is a strong inhibitor of the complement (C) system [5], [39]. In another study, Anderson and Johnson [40] showed by electron microscopy that leptospires belonging to saprophytic strain Patoc I and non-virulent serovar Canicola retained their shape but lost their outer sheath after incubation with complement and immune serum. In contrast, leptospires of the virulent serovar Canicola were not affected at all by complement and antibodies.

Incubation with either THP-1 cells or PBMC did not result in killing of any of the viable leptospiral strains. Indeed, previous studies reported similar findings [41], [42], suggesting that phagocytosis or the release of bactericidal mediators are not major processes involved in the killing of leptospires. An important conclusion on this observation is that caution is needed by interpretation of results obtained with stripped models for exploring cytokine responses upon leptospiral infection. Additionally, the data presented herein emphasize the importance to use viable low passage isolates to study the host response to *Leptospira* infection.

In conclusion, our results show that the innate human host response to leptospiraemia involve, but may not be limited to the action of TLR2, TLR4 and TLR5. Further research is needed on the mechanism of interactions of these TLRs to unravel their involvement during human leptospirosis.

## Supporting Information

Figure S1 **LigA westernblot.** Westernblot of whole lysates of leptospires detected with rabbit anti-LigA antibody as follows: lane 1 contains host-adapted serovar Lai type Langkawi, lane 2 host-adapted serovar Bataviae, lane 3 reference serovar Patoc strain Patoc I. Size markers are indicated to the left. The faint band of serovar Lai type Langkawi has largely been lost by the reproductions.
(TIF)

Figure S2 **Controls.** Human whole blood stimulation with different ligands in the absence or presence of TLR-inhibiting antibodies. Concentration of the ligands are as indicated. Anti-TLR antibodies were added at the following concentrations: 2500 ng/ml anti-TLR2, 1000 ng/ml anti-TLR4 and anti-TLR5. Polymixin B (PMB), 10 µg/ml, was added to compensate effects by residual free LPS in the various ligands. Significant cross-inhibition of blocking antibodies to heterologous TLRs did not occur (not shown).
(TIF)
