# Supplementary figures and images for: Potent Innate Immune Response to Pathogenic Leptospira in Human Whole Blood
Source: PLoS One. 2011 Mar 31;6(3):e18279. doi: 10.1371/journal.pone.0018279 (PMC3069077; doi:10.1371/journal.pone.0018279)

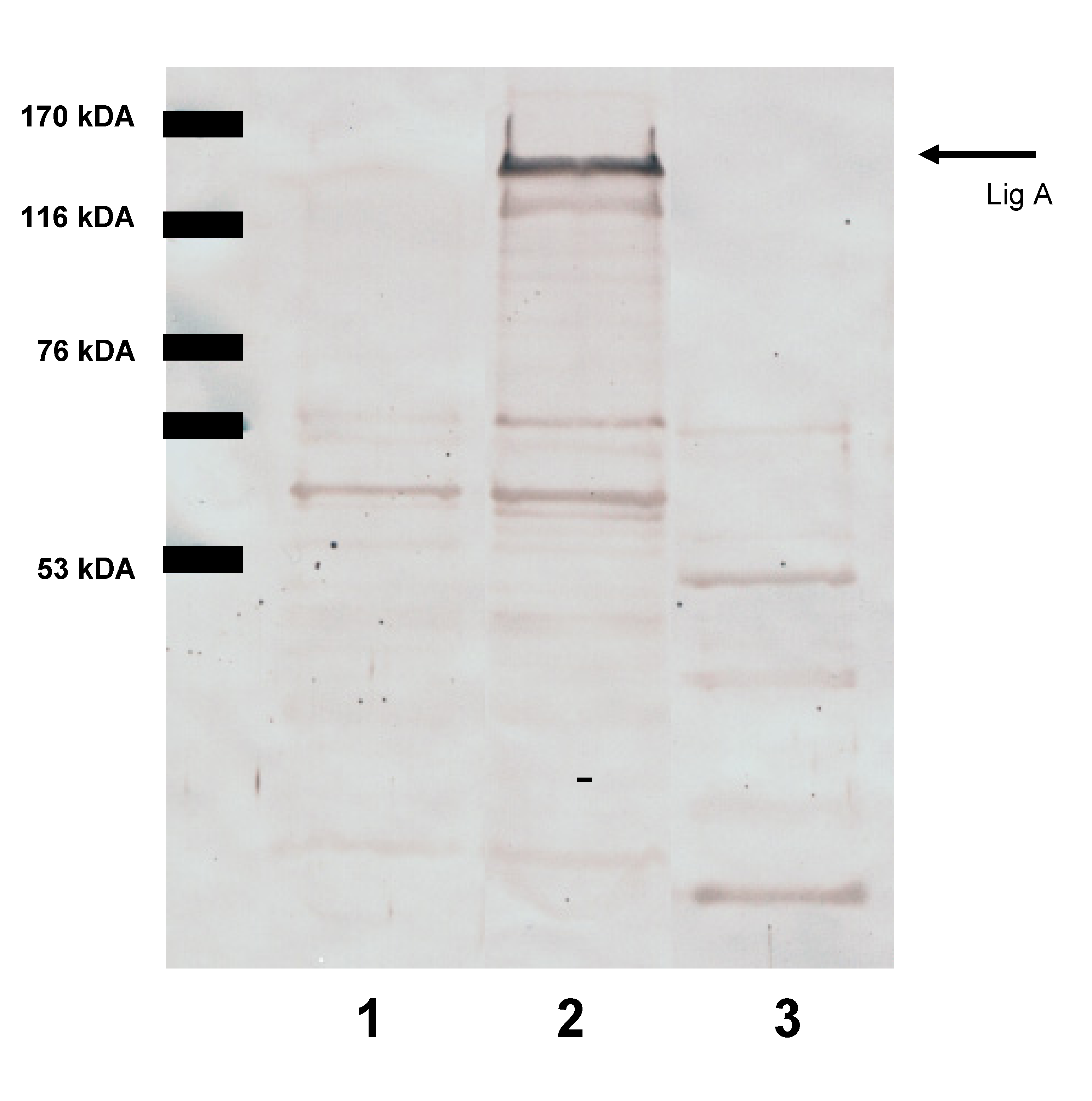

Supplement: Figure S1 — LigA westernblot. Westernblot of whole lysates of leptospires detected with rabbit anti-LigA antibody as follows: lane 1 contains host-adapted serovar Lai type Langkawi, lane 2 host-adapted serovar Bataviae, lane 3 reference serovar Patoc strain Patoc I. Size markers are indicated to the left. The faint band of serovar Lai type Langkawi has largely been lost by the reproductions. (TIF) [file pone.0018279.s001.tif]

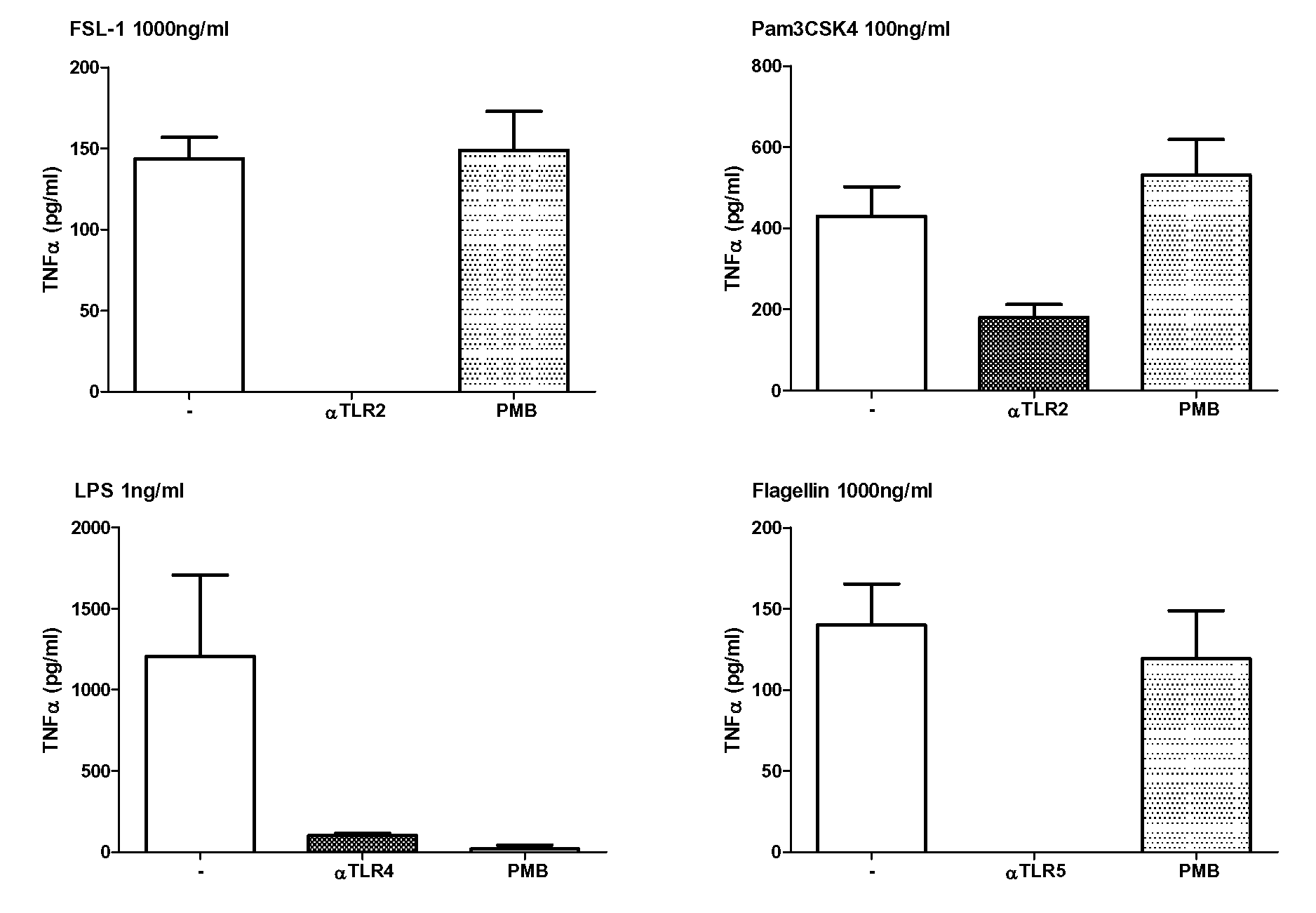

Supplement: Figure S2 — Controls. Human whole blood stimulation with different ligands in the absence or presence of TLR-inhibiting antibodies. Concentration of the ligands are as indicated. Anti-TLR antibodies were added at the following concentrations: 2500 ng/ml anti-TLR2, 1000 ng/ml anti-TLR4 and anti-TLR5. Polymixin B (PMB), 10 µg/ml, was added to compensate effects by residual free LPS in the various ligands. Significant cross-inhibition of blocking antibodies to heterologous TLRs did not occur (not shown). (TIF) [file pone.0018279.s002.tif]
